# Timing of ileocolic resection for Crohn's disease: A survey of the patient perspective in the ‘biological’ era

**DOI:** 10.1111/codi.70440

**Published:** 2026-04-01

**Authors:** Nilofer Husnoo, Ruchika Nongrum, Thomas Hall, Alexandra E. Whitman, Lynda Wyld, Alan J. Lobo, Jenna L. Morgan, Deborah Hawkins, Steven R. Brown, EBRIC Collaborators, S. R. Brown, R. Cooney, O. Faiz, A. Fawole, L. Ferrari, T. Grey, D. Hawkins, T. Hall, S. Henderson, N. Husnoo, J. Limdi, R. Nongrum, Z. Shamshudin, A. Shamsiddinova, A. E. Whitman, G. Williams, T. Wilson

**Affiliations:** ^1^ University of Sheffield Sheffield UK; ^2^ Sheffield Teaching Hospitals NHS Foundation Trust Sheffield UK; ^3^ Hull York Medical School Heslington UK; ^4^ Doncaster and Bassetlaw Teaching Hospitals NHS Foundation Trust Doncaster UK

**Keywords:** Crohn's disease, decision‐regret, ileocolic resection

## Abstract

**Background:**

Medical therapy is often favoured in the treatment of ileocaecal Crohn's disease (CD), with surgery often reserved for treatment failure. Data from the ‘pre‐biological’ era suggest that most patients who had an ileocolic resection for CD would have preferred to have surgery sooner. We revisited this observation in the current era and assessed decision‐regret relating to surgery.

**Methods:**

A multicentre cross‐sectional survey was conducted across nine centres in the United Kingdom with adult patients who had their first ileocolic resection for isolated terminal ileal or ileocolic CD in the 7 years preceding the study. Primary outcomes were patient preference for timing of resection and decision‐regret scale score (DRS).

**Results:**

Data from 171 completed surveys were analysed. Eighty (46.8%) patients were satisfied with the timing of surgery; 43.2% (74/171) wished they had surgery sooner and experienced a higher proportion of open procedures. The median DRS was 5/100. The only significant predictor of regret was a lower degree of involvement in shared decision‐making (SDM). Nearly half believed surgery to only be an option after all medical therapies had failed.

**Conclusion:**

The proportion who felt that their resection was left too late (43%) is still significant, despite being lower compared to the ‘pre‐biological’ era (75% in a previous similar study). Patients' regret relating to their first resection is generally low, especially when they are involved in SDM. However, SDM remains suboptimal. There is an urgent need for improved counselling and earlier surgical consideration in isolated ileocaecal CD, supported by multidisciplinary input.

**What does this paper add to the literature?**

In a study in the ‘pre‐biological’ era, most patients with a previous ileocolic resection for Crohn's disease would have preferred earlier surgery. Our contemporary data show that a smaller but significant proportion (>40%) still wish they had surgery sooner, highlighting a need for shared decision‐making regarding optimal timing of surgery.

## INTRODUCTION

In the early 1990s, a study of patients with a previous ileocolic resection for Crohn's disease (CD), conducted by Scott and Hughes, showed that three‐quarters would have preferred to have surgery sooner [1]. Reasons for this included the severity of symptoms pre‐operatively, improved well‐being post‐operatively and being in drug‐free remission after their resection. This suggested that surgery was being left too late in the treatment of ileocaecal CD. This survey was conducted before the ‘biological era’ when medical options were more limited, less effective, often included steroids and had a different adverse event profile to the modern era.

The therapeutic landscape for CD has evolved considerably. Experience in the use of advanced therapy including biologics has accumulated through strategies such as early and top‐down treatment, dose optimisation and treat‐to‐target approaches [2, 3]. On the other hand, surgical techniques for CD have also improved, and surgery is safer with lower morbidity [4, 5]. Evidence in favour of earlier surgery, particularly for isolated terminal ileal or ileocolic disease, is growing [6, 7, 8]. Performing a resection sooner as an alternative to medical therapy, instead of considering surgery as a ‘last resort’ when medical options have been exhausted, appears to lead to more stable remission with a reduced need for medication and subsequent surgery in the long term [6].

However, a recent UK‐based study suggests that surgery is still performed late in the disease course [9]. Persevering with medical treatment in a bid to avoid surgery may seem like an attractive notion, especially with the range of medical options available. Long waiting lists for elective procedures are also likely to contribute to delayed surgery [9]. This may come at a cost to the patient's quality of life and may not necessarily align with patients' wishes, as previously shown [1].

The aim of this study was to re‐evaluate the patient perspective on the timing of their first ileocolic resection for isolated terminal ileal or ileocolic CD in the current ‘biological’ era. We also aimed to assess patients' decision‐regret relating to their first resection, and how this relates to pre‐ and peri‐operative treatment experiences and post‐operative outcomes.

## METHODS

A multicentre cross‐sectional patient survey was conducted between February and December 2024 across nine centres in England and Wales (4 secondary and 5 tertiary care centres for inflammatory bowel disease (IBD)). Ethical approval was provided by the London Brent Research Ethics Committee (Ref: 23/PR/0568). The study has been reported in line with CROSS (consensus‐based checklist for reporting of survey studies). The protocol has been described previously and will be summarised here [10].

### Survey design, validation and content

Items for the survey questionnaire were generated from themes identified from a literature review [6] and a qualitative study of healthcare professionals with experience in treating IBD [9], with input from clinician and patient experts, ensuring content validity. Face validity, comprehension and acceptability were evaluated by pilot‐testing the questionnaire with three patients meeting the eligibility criteria at one participating centre. The questionnaire was modified based on their feedback; the final version has been provided as Supporting Material (S1). A paper and an electronic version (hosted on Qualtrics®) were produced, with an accompanying information sheet about the study. Paper and electronic formats were chosen over telephone interviews to maintain anonymity and allow completion in patients' own time, given the survey length and sensitive nature of some questions.

### Outcomes

The primary outcomes were patient preference for timing of resection and decision‐regret scale score (DRS) relating to the decision to have surgery (described in Table 1) [11]. The secondary outcomes were explanatory factors that may predict regret.

**TABLE 1:** Tools used in the survey to evaluate patient‐reported measures.

Tool	Construct measured	Interpretation
Decision‐regret scale	Validated 5‐item scale that measures remorse after a healthcare‐related decision	0 represents no regret and 100 represents high regret. A DRS threshold of 25 is deemed indicative of strong regret (i.e. scores of 0–25 represent mild or no regret [12, 13, 14])
Body image scale (BIS)	Based on the Hopwood Body Image Scale [15]. A modification, which excludes the last question about surgical scar, has been validated for use in IBD patients to assess body image [16].	On a scale of 0–27, a lower score implies better body image.
Cosmetic score	Score measuring satisfaction with surgical scar(s) using a scale previously used in studies of patients who had an ileocolic resection for CD [7, 17]	On a scale of 3–24, a higher score indicates greater satisfaction with the scar
CollaboRATE tool	3‐item validated patient‐reported measure of shared decision‐making (SDM) used to assess the quality of the decision‐making process [18]	A CollaboRATE mean score is the mean of the scores for the 3 items. On a scale of 0–9, a higher score indicates a higher level of SDM. A CollaboRATE ‘top score’ (each of the 3 items rated with the highest score), indicates the highest level of patient satisfaction with SDM.

### Covariates

Data were collected on baseline participant characteristics, pre‐operative treatment, patients' understanding of the role of surgery in their treatment, post‐operative outcomes and patient‐reported measures of shared decision‐making (SDM), body image and satisfaction with surgical scar(s). An overview of the tools used in this survey to collect data on patient‐reported measures is provided in Table 1; they are incorporated in questions Q2.17–Q2.22 of the questionnaire (S1). Data for all variables were patient‐reported (medical records were not accessed), except for centre type (which was confirmed by the principal investigator at each site).

### Eligibility, sampling and recruitment

Patients, aged 18 and above, were included if they had their first ileocolic resection or right hemicolectomy, with or without an anastomosis, for CD localised to the terminal ileum or ileocaecum (confirmed using clinical records), in the 7 years preceding the launch of the study. This study period was chosen to capture current experiences, minimising bias due to temporal variations in surgical practice and recall bias. We excluded patients who had a resection performed as an emergency for their first presentation of CD, patients diagnosed with CD incidentally through a resection for an alternative pathology, and patients whose surgery was less than 3 months before recruitment to allow time for recovery.

The target population at each participating centre was identified from hospital databases. Convenience sampling was employed. Paper questionnaires were posted or a link to the online survey was emailed, the latter being the favoured recruitment approach; patients could also be approached in hospital by clinicians or research teams. Each patient contacted was allocated a unique ID to pseudonymise questionnaires. Non‐respondents received up to two postal or email reminders with a telephone reminder in between to improve response rates. No patient‐identifiable data were collected. The Qualtrics® database was only accessible by the central research team; paper questionnaires were digitised locally and emailed to the central research team and were not accessible by the patients' care teams, ensuring participants' anonymity and confidentiality.

The original target sample size was based on the estimated number of eligible patients across all sites [10]; this was found to have been overestimated after the launch of the study. We revised the calculation to detect a 10‐point difference in DRS across timing preference groups with 80% power (α = 0.05). The minimum required sample size was 112 (one‐way ANOVA). Allowing for item‐level non‐response, the target sample size was 135 participants.

### Data analysis

Statistical analysis was performed using IBM SPSS v29. Continuous variables were presented as medians with interquartile ranges (IQR), compared using the Wilcoxon rank‐sum or Kruskal–Wallis test, and associations with other continuous variables evaluated using Spearman's rank correlation. Categorical variables were compared across groups using Pearson's χ^2^ test. Linear regression identified factors predicting decision‐regret. Exploratory analyses comparing patients who had emergency surgery or a stoma with patients who had elective surgery and a primary anastomosis (to mirror the cohort in Scott and Hughes's study) were done to ascertain differences in timing preferences and regret. Records with missing data for the variables of interest were excluded. Free‐text responses were thematically analysed.

## RESULTS

**FIGURE 1:**
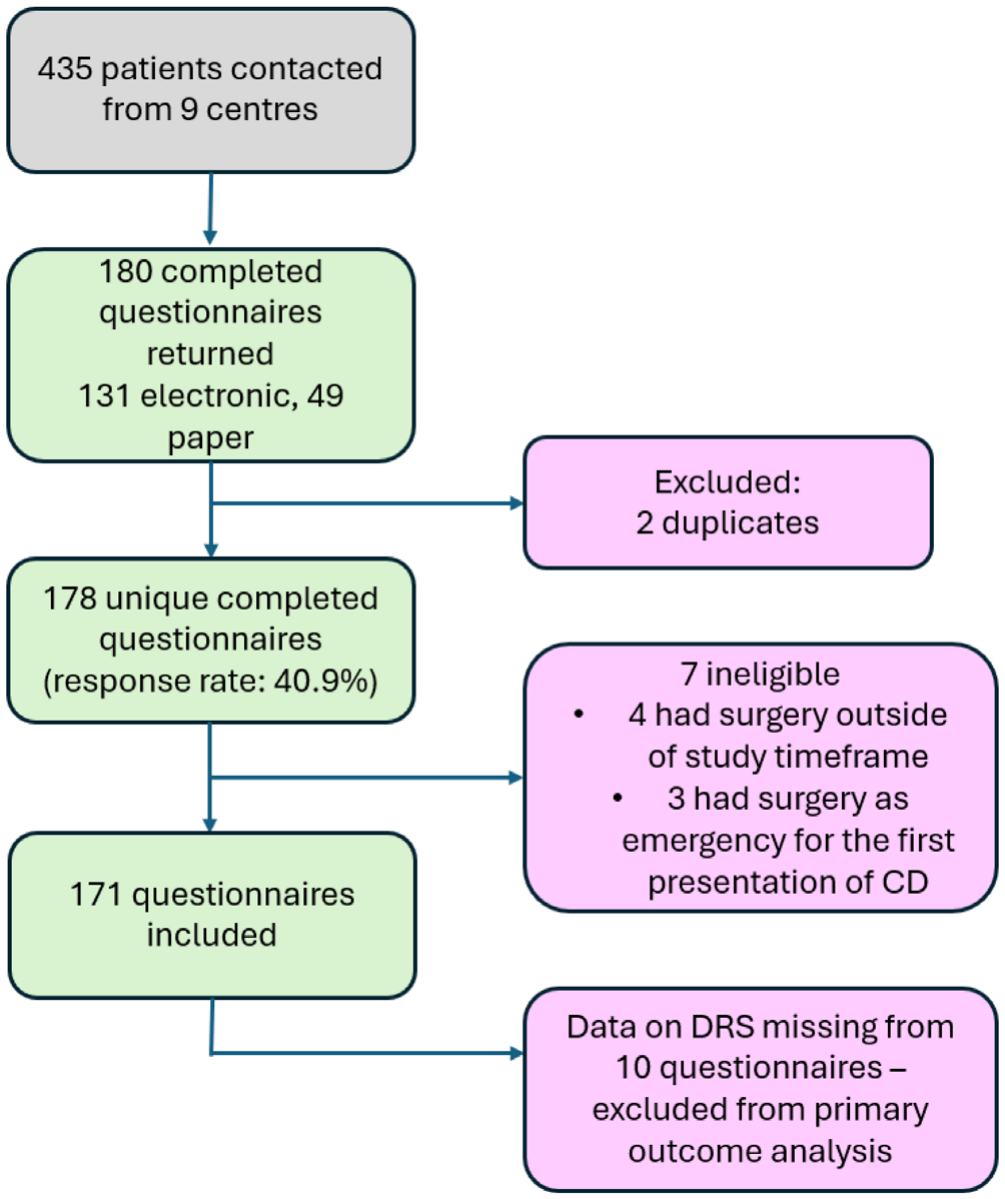
Flow diagram of patient recruitment and eligibility. CD, Crohn's disease; DRS, decision‐regret scale score.

The survey was completed by 178 patients (response rate of 40.9%, 178/435). Seven were ineligible and were excluded; data from 171 questionnaires were analysed (Figure 1).

### Participant, disease and treatment characteristics

**FIGURE 2:**
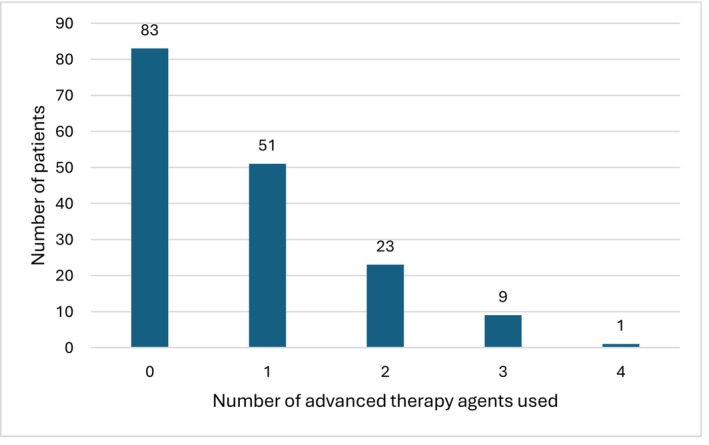
Advanced therapy use between diagnosis and surgery (missing data: *N* = 4).

Participant characteristics are shown in Table 2. The median times since diagnosis of CD and since the first ileocolic resection at the time of survey completion were 108 months (IQR 66–192) and 52 months (IQR 30–68), respectively. The first resection was performed a median of 55 months (IQR 14–156) after diagnosis. Most patients were from tertiary referral centres (110/171, 64.3%). Figure 2 illustrates the number of advanced therapy agents used before surgery – 19.8% (33/167) trialled more than one biologic before surgery.

**TABLE 2:** Participant characteristics.

	Number of patients (%) Total *N* = 171
**Level of IBD care provided by participating centre**	
Secondary care	61 (35.7)
Tertiary care	110 (64.3)
**Gender**	
Male	76 (44.4)
Female	95 (55.6)
**Ethnicity**	
White British	152 (88.9)
Asian/Asian British	9 (5.3)
Mixed/Multiple ethnic groups	4 (2.3)
Any other white background	3 (1.8)
Other	2 (1.2)
Black/African/Caribbean/Black British	1 (0.6)
**Highest level of education**	
One or more GCSEs	53 (31.0)
One or more A‐levels	39 (22.8)
Bachelor's degree (e.g. BSc, BA)	38 (22.2)
Postgraduate degree (Masters/MD/Doctorate)	15 (8.8)
No qualifications	11 (6.4)
Other	15 (8.8)
**Experience of drug treatment between diagnosis and surgery** ^a^	
5‐ASA	20 (11.7)
Advanced therapy (including biologics and oral small molecules)	88 (51.5)
Methotrexate	17 (9.9)
Thiopurine	80 (46.8)
Steroids	109 (63.7)
Liquid diet	27 (15.8)
No experience of medication	24 (14.0)
**Urgency of procedure** ^b^	
Planned	134 (78.4)
Elective	88 (51.5)
Semi‐elective	46 (26.9)
Emergency	37 (21.6)

Abbreviations: IBD, inflammatory bowel disease; IQR, interquartile range.

^a^ Totals exceed 171 due to individual participants having experience of more than one medical treatment.

^b^ Elective defined as ‘planned in advance and put on a waiting list’, semi‐elective defined as ‘urgent, but admission planned and arranged within days to a few weeks’, emergency defined as ‘performed urgently during an unplanned admission’.

### Understanding of the role of surgery

Of 170 patients, 38 (22.4%) were unaware of surgery as an option when treatment for CD was first discussed. Most (67.5%, 114/171) first learnt about surgery from a gastroenterologist, either exclusively or alongside other sources. Nearly half (46.4%, 78/168) thought that surgery was an option “after they had tried all possible drugs” and only 11.3% (19/168) were aware that it could be considered as an alternative to medical therapy (complete results in Table S1).

### Surgical procedures and outcomes

As shown in Table 3, emergency resections were performed in 21.6% (37/171) of participants, 59.4% (101/170) had an open procedure, 26.3% (45/171) had a stoma formed at the time of the index procedure, and 58.8% (100/170) reported at least one surgical complication. At the time of survey completion, around half reported post‐operative disease recurrence, 5.8% (10/171) had a further resection and 53.8% (92/171) were on advanced therapy. Patient‐reported measures are summarised in Table 4.

**TABLE 3:** Surgical outcomes.

	Number (%)
**Approach to surgery** ^a^	
Open	101 (59.4)
Laparoscopic	69 (40.6)
**Stoma formation as part of procedure, or during the same admission**	
Yes	45 (26.3) 21 (12.3) had a stoma at the time of survey completion
No	126 (73.7)
**Complications** ^b^	
No reported complications	70 (41.2)
*Short‐term complications*	
Anastomotic leak	10 (5.9)
Wound problem (infection/dehiscence)	32 (18.8)
Emergency return to theatre	8 (4.7)
VTE	3 (1.8)
*Long‐term complications*	
Hernia	23 (13.5)
Bile acid malabsorption	40 (23.5)
**Disease recurrence**	
Yes	89 (52.0)
Repeat resection during study period	10 (5.8)
No	82 (48.0)
**Drug treatment at the time of survey completion (snapshot of ‘current’ treatment)** ^c^	
5‐ASA	4 (2.3)
Advanced therapy (including biologics and oral small molecules)	92 (53.8)
Methotrexate	5 (2.9)
Thiopurine	32 (18.7)
Steroids	6 (3.5)
Liquid diet	0 (0)
No medication	56 (32.7)

Abbreviations: ASA, aminosalicylic acid; VTE, venous thromboembolism.

^a^ Missing data: *n* = 1.

^b^ Missing data: *n* = 3.

^c^ Totals exceed 171 due to some participants having more than one medical treatment.

**TABLE 4:** Patient‐reported measures.

Patient‐reported measures	Score
Median body image score^a^ (range, IQR)	5.0 (0–27, 10.0)
Median cosmetic score^b^ (range, IQR)	17.0 (3–24, 8.0)
Median CollaboRATE mean score^c^ (range, IQR)	7.67 (0.33–9.00, 3.00)
CollaboRATE top score^c^ (range, IQR)	27.3% (*n* = 45/165)

^a^ Missing data *n* = 7.

^b^ Missing data *n* = 2.

^c^ Missing data *N* = 6.

### Outcomes

#### Preference for timing of surgery

Of the 171 patients, 43.2% (74/171) wished they had surgery sooner, by a median of 11 months (range 1–120), while 80 (46.8%) felt it occurred at the right time. Seventeen patients (9.9%) wished it was later (*n* = 2) or that they never had it (*n* = 15); these two groups were combined for subsequent analyses. Table 5 presents a thematic analysis of respondents' justifications for their choice, alongside representative quotes.

**TABLE 5:** Participants' thoughts on the timing of their first ileocolic resection.

Reasons cited for this	Representative quotes
**‘I wish I had the operation sooner’**	***N* = 74 (43.2%)**
Severity of symptoms	‘if I had the operation sooner could have got on with my life; instead, I spent years in pain’
Significant improvement in health and quality of life following surgery	‘my health and quality of life improved significantly following the first surgery’
Disease progression attributed to delayed surgery	‘If I was listened to sooner, the amount of bowel removed would have been less’.
Challenging peri‐operative experience and recovery attributed to delayed surgery	‘I needed to be in hospital for about 3 weeks prior to surgery. I was put on TPN for about 2 of those weeks’. ‘I could have had it done via keyhole which would have avoided wound complications’.
Recurrent hospital admissions	‘being admitted to A&E more times than I can remember – in severe pain’.
Ineffective medical treatments	‘I wouldn't have needed to trial so many different biologics and steroids’.
**‘I had the operation at the right time’**	***N* = 80 (46.8%)**
Complications necessitated surgery	‘I had developed a fistula between my bladder and my bowel’. ‘…no option as I had a blockage in my bowel’.
Medical options had been exhausted	‘Left to last resort as only moves somewhere else’. ‘I had tried several types of medication and none worked long term’.
Done in preventative window	‘My bowel was getting gradually worse and any later could have potentially resulted in a stoma’.
**‘I wish I had the operation later’**	***N* = 2 (1.2%)**
No opportunity to trial medication	‘No opportunity to trial out other medication to suppress the inflammation pre‐operation’.
**‘I wish I never had the operation’**	***N* = 15 (8.8%)**
Could have been avoided with optimisation of medical treatment	‘If I had received more proactive care […] I would not have needed the surgery’.
Disease recurrence	‘It only made a difference for a short period of time’.
Long‐term consequences of complications	‘…left with a huge swelling in my stomach [hernia]…I'm feeling extremely depressed with it as I've always had a flat stomach’.
Symptoms worse (diarrhoea)	‘Since the op I have chronic diarrhoea…No one warned me of this!…It's ruined my quality of life’.

#### Factors associated with timing preferences

Open surgery was less common among patients satisfied with surgical timing (38/79, 48.1%) compared to those who wished they had surgery earlier (49/74, 66.2%) or later/never (14/17, 82.4%) (*p* = 0.009). When comparing the group that was satisfied with the timing of surgery and those who wish they had it later/never, the former had a better body image and greater satisfaction with surgical scars and experienced a higher degree of SDM (median BIS score 4.0 vs. 9.0, *p* = 0.036, cosmetic score 18.0 vs. 10.0, *p* = 0.027 and CollaboRATE mean score 8.0 vs. 6.0, *p* = 0.047, respectively). These scores did not differ significantly between the ‘satisfied’ group and those who wished they had surgery sooner. There was no other difference in baseline characteristics or surgical outcomes across the three groups.

Considering open procedures (as the only baseline characteristic associated with timing preferences), they were found to be more common in the emergency setting than in the planned setting (32/37, 86.5% vs. 69/133, 51.9%; *p* < 0.001) but were not associated with other baseline variables. They were also associated with worse body image and cosmetic score compared with laparoscopic procedures (median BIS score 7.0 vs. 3.0, *p* < 0.001 and median cosmetic score 15.0 vs. 20.0, *p* < 0.001, respectively).

#### Decision‐regret scale score relating to decision to have surgery

**FIGURE 3:**
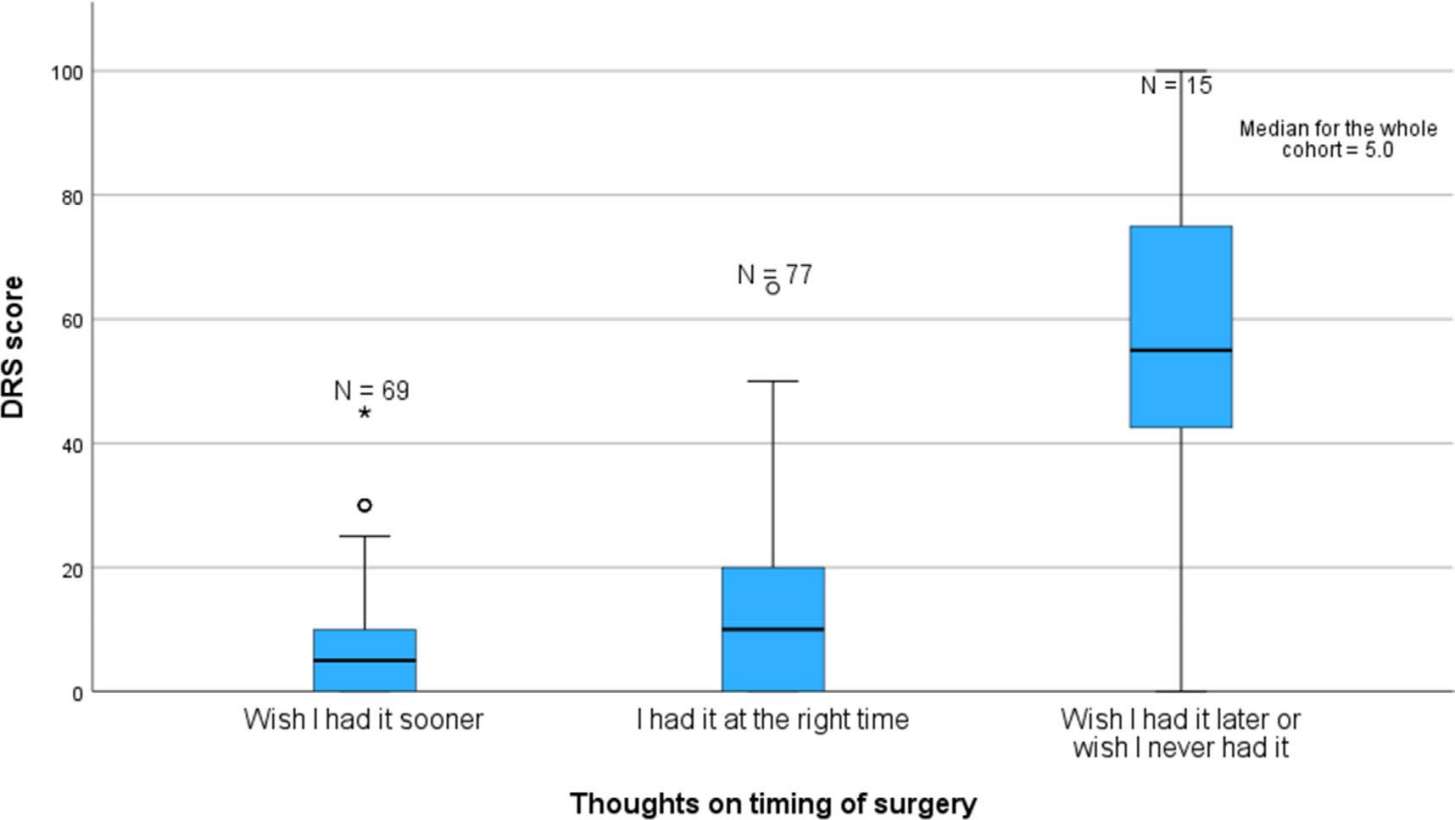
Median decision regret scale scores according to patients' preferences for timing of surgery. DRS: Decision‐regret scale score. Missing data: *N* = 10. ‘Wish I had it sooner’ median score 5.0 (0–45, IQR 10.0); ‘I had it at the right time’ median score 10.0 (0–65, IQR 22.5), ‘Wish I had it later or Wish I never had it’ median score 55.0 (0–100, IQR 35.0). *p* < 0.001.

The overall median DRS was 5.0 (0–100, IQR 25.0). It varied significantly across timing preference groups (Figure 3, *p* < 0.001), with patients who wished they never had surgery or had it later reporting substantially higher regret (median 55.0) compared with those satisfied with timing (median 10.0) or who wished surgery was sooner (median 5.0) (*p* < 0.001). Regret was positively correlated with a higher BIS score (worse body image) (*r*_*s*_ = 0.201, *p* = 0.012) and negatively correlated with cosmetic score (*r*_*s*_ = −0.191, *p* = 0.016) and CollaboRATE mean score (*r*_*s*_ = − 0.278, *p* < 0.001). No other variables were associated with decision‐regret.

#### Predictors of regret

Simple linear regression identified five variables that were significantly associated with decision‐regret: open (vs laparoscopic) surgery (β 7.344, 95%, CI 0.936–13.752, *p* = 0.025), the occurrence of post‐operative complications (β 7.759, 95%, CI 1.384–14.134 to −1.384, *p* = 0.017), a lower CollaboRATE mean score (β −2.506, 95%, CI −3.903 to −1.108, *p* < 0.001), a higher BIS score (i.e. poorer body image) (β 0.807, 95% CI 0.366–1.247, *p* < 0.001) and a lower cosmetic scale score (β −0.883, 95% CI −1.431 to −0.334, *p* = 0.002). Age, centre type, gender, disease duration before surgery, pre‐operative medication, number of advanced therapy agents used before surgery, urgency of operation, stoma formation, disease recurrence, repeat resection and post‐operative medication use were not significant predictors. Multiple linear regression (including the five variables above) identified only CollaboRATE mean score as a significant predictor of decision‐regret (β −2.082, 95% CI −3.495 to −0.669, *p* = 0.004).

#### Subgroup analysis of planned surgery with a primary anastomosis

Of 112 patients who had a planned procedure with a primary anastomosis, 46.4% felt it occurred at the right time (52/112), the same number wished they had it sooner, and 7.1% (8/112) wished they never had it or had it later. The median DRS was 5 (IQR 30). Timing preferences and DRS did not differ significantly when compared to those who had emergency procedures and/or a stoma.

## DISCUSSION

This study offers contemporary insights into patient perspectives on the timing of ileocolic resection for Crohn's disease (CD). The main finding is that fewer patients expressed a preference for earlier surgery compared with the landmark study by Scott and Hughes (43% vs. 75%). Despite this, the regret associated with the decision to have surgery was low.

Methodological differences between the studies do not fully explain the lower proportion preferring earlier surgery. The previous study only included patients who had planned procedures with an anastomosis; we also included patients who had emergency surgery and/or a stoma. However, excluding the latter group from the analysis did not impact timing preferences or decision‐regret. Repeat resections were included in the previous survey while we only included first‐time resections. As patients are less likely to prefer surgery sooner when having a second resection [1], this is also unlikely to account for the difference in our findings. Notably, the therapeutic landscape has evolved considerably since the original study. Many advanced therapy agents are now available which have improved disease control and quality of life [19, 20, 21, 22, 23]. The increased availability of these agents likely reduces the urgency for surgery, possibly explaining the lower proportion favouring earlier resection now. The level of satisfaction with surgical timing in our cohort mirrors that found in a recent European study with IBD patients [24].

Nevertheless, the fact that 43% of patients would have preferred earlier surgery is important. Explanations that emerged centred on themes of debilitating symptoms, multiple hospital admissions and disease progression, with substantial improvement in quality of life post‐operatively— suggesting that, in many, surgery is performed late in the disease course. Notably, 1 in 5 trialled two or more advanced therapy agents before surgery. Conversely, those satisfied with surgical timing valued ‘having the opportunity’ to trial medication before surgery, while still having surgery in a ‘preventative window’ before significant deterioration. This highlights the challenge of identifying the optimal time for surgery in the context of rapidly expanding medical options, underscoring the need for a close working relationship between gastroenterology and surgical teams to improve surgical decision‐making and reduce delays and for local services to be set up to minimise waiting times for surgery.

The low decision‐regret (median of 5/100) is consistent with IBD surgery literature [25, 26], and the broader literature [12]. The reported regret was low despite half reporting disease recurrence and two‐thirds being on medication at the time of the survey. Our thematic analysis suggests that this likely reflects improved symptom‐control and quality of life, which tend to be patients' primary therapeutic goals [27, 28]. The 9% who wished surgery was delayed or that they never had it, had a much higher decision‐regret (median 55/100), the main cited reasons being disease recurrence and long‐term surgical complications. In fact, around a quarter of participants reported bile acid diarrhoea—a common and underdiagnosed consequence of ileal resection that significantly impairs quality of life and requires active management [29, 30].

The only significant predictor of regret was a lower degree of SDM. Patients satisfied with surgical timing also reported better SDM scores than those who wished they had surgery later or never had it. In recognition of the benefits of SDM especially in improving patient satisfaction, policy recommendations advocate for healthcare professionals to actively engage patients in decisions about their care [31, 32]. Yet, only 27% of our patients achieved a CollaboRATE top score, indicating “gold standard” SDM. Key to SDM is empowering patients to consider all their options. However, nearly half in our cohort believed surgery was only an option when all drugs had failed; only 1 in 10 appreciated its role as an alternative to medical therapy. With evidence and guidelines increasingly supporting surgery as an alternative to medical therapy in localised ileocolic disease [7, 33, 34], patients require adequate counselling about early surgery to make an informed choice. Earlier involvement of surgeons as a source of information in this predominantly medically managed group may provide a more balanced view of treatment options [5, 9, 24].

Open procedures were more common among patients dissatisfied with surgical timing compared to those who were satisfied. The latter also reported better body image and cosmetic scores, likely reflecting the greater proportion of minimally invasive procedures. Even though our 40% rate of laparoscopy aligns with other international cohorts when converted cases are counted as open [35], this proportion is still low, especially considering that two‐thirds of our cohort were patients at a tertiary referral centre. Our findings suggest that many procedures are being performed for advanced disease, reinforcing the idea that timely referral and intervention could facilitate minimally invasive approaches, which offer reduced incisional hernia rates and fewer postoperative complications [36]. Open procedures were also more common in the emergency setting. Emergency resections were done in 22% of our cohort—these are also likely to represent procedures performed for advanced disease. Emergency resections increase complication and mortality rates in CD and should ideally be avoided [33, 37, 38, 39].

This study has some limitations. The response rate was modest, though comparable to similar studies [26, 40]. Patients with strongly positive or negative experiences may have been more motivated to respond, somewhat limiting generalisability. Resource constraints did not allow us to investigate the characteristics of non‐respondents. Reliance on patient recall introduces potential bias. However, given the significance of a patient's first resection for CD, patient panel feedback suggested that recall was likely to be accurate; our primary aim was also to measure patient‐reported outcomes rather than clinical parameters. The study period also includes the COVID‐19 pandemic, when healthcare delivery and elective work were affected. However, an analysis of procedures performed during the pandemic revealed no significant differences in key outcomes compared to non‐pandemic periods (Table S2). Strengths of the study include its large sample size compared to other surveys in similar populations [26, 40, 41], inclusion of both elective and emergency procedures, involvement of patients from diverse centres across England and Wales, and representation of a broad range of surgical experiences including stoma formation. Unlike prior studies, this is the first to systematically evaluate decision regret and other patient‐reported measures in patients undergoing their first ileocolic resection for CD, providing a unique and timely patient‐centred perspective.

## CONCLUSION

Although fewer patients would prefer an earlier ileocolic resection in the ‘biological era’ than in previous studies, over 40% would have preferred earlier surgery. Many procedures are probably being done for advanced disease, reflected by high rates of open and emergency surgery. Decision‐regret relating to surgery is generally low, especially when patients are involved in shared decision‐making. However, shared decision‐making remains suboptimal, with many patients unaware of surgery as an alternative therapeutic pathway. These findings underscore the need for improved counselling and earlier surgical consideration in isolated ileocolic CD, supported by multidisciplinary care pathways that promote optimal timing and technique.

## AUTHOR CONTRIBUTIONS

**Nilofer Husnoo:** Conceptualization; data curation; formal analysis; funding acquisition; investigation; methodology; project administration; writing – original draft; writing – review and editing. **Ruchika Nongrum:** Formal analysis; writing – review and editing. **Thomas Hall:** Data curation; investigation; writing – review and editing. **Alexandra E. Whitman:** Conceptualization; writing – review and editing. **Lynda Wyld:** Conceptualization; methodology; supervision; writing – review and editing. **Alan J. Lobo:** Conceptualization; funding acquisition; methodology; supervision; writing – review and editing. **Jenna L. Morgan:** Conceptualization; methodology; supervision; writing – review and editing. **Deborah Hawkins:** Data curation; investigation; project administration; writing – review and editing. **Steven R. Brown:** Conceptualization; funding acquisition; methodology; supervision; writing – review and editing. All authors revised the article for important intellectual content, provided final approval of the version to be published, and agreed to be accountable for all aspects of the work.

## FUNDING INFORMATION

The study is funded by a grant from Crohn's and Colitis UK awarded to NH.

## CONFLICT OF INTEREST STATEMENT

AJL receives speaker and/or consulting fees for Takeda, BMS, Abbvie, Medtronic, Vifor, Janssen, Sandoz, MSD, Pfizer and Shield Therapeutics. The study funder has had no role in the study design and writing of the report.

## ETHICS STATEMENT

Ethical approval to conduct the study has been provided by the London‐Brent REC (ref 23/PR/0568).

## CLINICAL TRIAL REGISTRATION

Clinicaltrials.gov database (NCT06116604).

## Supporting information


Table S1.

## Data Availability

The data that support the findings of this study are available from the corresponding author upon reasonable request.
