# Supplementary material for: Timing of ileocolic resection for Crohn's disease: A survey of the patient perspective in the ‘biological’ era
Source: Colorectal Dis. 2026 Apr 1;28(4):e70440. doi: 10.1111/codi.70440 (PMC13044388; doi:10.1111/codi.70440)
Supplement: Supplementary file 1 — Table S1. [file CODI-28-0-s001.docx]

## EBRIC collaborators

Brown SR^1^, Cooney R^2^, Faiz O^3^, Fawole A^4^, Ferrari L^5^, Grey T^6^, Hawkins D^1^, Hall T^1^, Henderson S^6^, Husnoo N^1,7^, Limdi J^8^, Nongrum R^1,7^, Shamshudin Z^4^, Shamsiddinova ^3^, Whitman AE^9^, Williams G^10^, Wilson T^11^

1: Sheffield Teaching Hospitals NHS Foundation Trust

2: University Hospitals Birmingham NHS Foundation Trust

3: London North West University Healthcare NHS Trust

4: Mid Yorkshire Hospitals NHS Foundation Trust

5: Guy’s and St Thomas’ NHS Foundation Trust

6: Calderdale and Huddersfield NHS Foundation Trust

7: University of Sheffield

8: Northern Care Alliance NHS Foundation Trust

9: Hull York Medical School

10: Aneurin Bevan University Health Board

11: Doncaster and Bassetlaw Teaching Hospitals NHS Foundation Trust

## Questionnaire administered in study

Before starting the survey, please confirm that you: are 18 years of age or older, have read all of the information above, and consent to taking part in the study.

- I consent and wish to participate in the study.
- I do not consent and do not wish to participate in the study.

**Section 1: Please tell us about yourself**

1. How old are you? Please write your age in years __________
2. Please indicate your gender (tick one of the boxes):

- Male
- Female
- Other Please specify __________________
- Prefer not to answer

1. How long has it been since you were diagnosed with Crohn’s disease? Please state in years and months __________________
2. Please tell us about your highest level of education (tick one of the boxes):

- I have one or more GCSEs
- I have one or more A-levels
- I have a Bachelor’s degree (e.g. BSc, BA)
- I have a post-graduate degree (Masters/ MD/ Doctorate)
- I do not have any qualifications
- Other

1. Which ethnic group do you belong to?

- **White British**
- **Any other White background**
- **Mixed/Multiple ethnic groups**
- **Asian/Asian British**
- **Black/ African/Caribbean/Black British**
- **Other ethnic group – please specify ______________________**
- **Prefer not to answer**

**Section 2: This section is about your FIRST operation to remove the part of the bowel affected by Crohn’s disease. Your clinician may have described the operation using any of the following terms: terminal ileal resection, ileocaecal resection, ileocaecectomy, right hemicolectomy, limited right hemicolectomy, right colectomy, limited right colectomy.**

**You may have had more than one operation to remove bowel in this area, but unless otherwise specified, we want to know about the first operation you had.**

1. How long ago was your first operation to remove the part of the bowel affected by Crohn’s disease? Please state in years and months ______________________
2. What treatment were you on or had you tried for Crohn’s disease before this operation? Please tick all that apply:

- **No medication**
- **Liquid diet**
- **Steroids**
- Azathioprine
- **Methotrexate**
- Biologics (i.e. infusions or injections such as infliximab/ adalimumab/ vedolizumab/ ustekinumab)
- Sulfasalazine
- **Other – please specify _____________________**

1. If you were on a biologic before your operation, how many different types of biologics did you try (before your operation)? _______________

(If you did not try any biologic before your operation, write “0”)

1. Was it a planned (elective) operation, or was it an emergency operation?

- **Elective (planned in advance and put on a waiting list)**
- **Emergency (performed urgently during an unplanned admission)**
- **Semi-elective (urgent, but admission planned and arranged within days to a few weeks)**

1. **Was the operation done keyhole or open?**

- **Keyhole (laparoscopic)**
- **Open**

1. **Was a stoma formed at the time of this operation, or during the same admission?**

- **Yes**
- **No**

1. **Do you have a stoma now?**

- **Yes**
- **No**

1. **Did you have any complication after your operation? Tick all that apply.**

- **No complication**
- **Problem with the join formed in the bowel (also called a ‘leak’)**
- **Bleeding**
- **Wound problem (infection, breakdown)**
- **Other infection (chest infection, urine infection)**
- **I was taken back to theatre for a second operation during the same admission**
- **I have developed a hernia since the operation**
- **Bile acid malabsorption**
- **Other – please specify ___________________________**

1. **Has your Crohn’s come back in the bowel since this operation?**

- **Yes**
- **No**

1. **Have you had a second operation to remove some more bowel as a result of the disease coming back?**

- **Yes**
- **No**
  1. **Are you currently taking any medication for Crohn’s disease?**
- **No medication**
- **Liquid diet**
- **Steroids**
- Azathioprine
- **Methotrexate**
- Biologics (i.e. infusions or injections such as infliximab/ adalimumab/ vedolizumab/ ustekinumab)
- **Other – please specify _____________________**
  1. **Thinking about the timing of your first operation** **to remove the part of the bowel affected by Crohn’s disease**, which of the following statements applies?

Tick **one** box and explain the reason(s) for your answer.

- **I wish I had the operation sooner because ________________________________ ______________________________________________________________________________________________________________________________________________________________________________________________________**

**Please go to question 2.13.**

- **I wish I had the operation later because _________________________________ ______________________________________________________________________________________________________________________________________________________________________________________________________**

**Please go to question 2.13.**

- **I had the operation at the right time because _____________________________ ____________________________________________________________________________________________________________________________________**

**Please go to question 2.14.**

- **I wish I never had the operation because ________________________________ ______________________________________________________________________________________________________________________________________________________________________________________________________**

**Please go to question 2.14.**

- 1. Please state how many **months** sooner or later you wish you had the operation.
- **I wish I had the operation sooner and I would have liked to have my operation _________ months sooner.**
- **I wish I had the operation later and I would have liked to have my operation _________ months later.**
  1. Were you aware that surgery was a treatment option for your type of Crohn’s disease when treatment options for Crohn’s were first discussed with you?
- Yes
- No
  1. How did you first learn about surgery as a treatment option? Tick all that apply.
- **From a gastroenterologist**
- **From a surgeon**
- **From an inflammatory bowel disease nurse specialist**
- **From a friend/ family/ relative**
- **From the internet**
- **Other – Please specify _______________________________________**
  1. Which of the following statements describes your views on surgery for Crohn’s disease **BEFORE** you were listed for your first operation?
- I was not aware that surgery was a treatment option for me before I was offered or listed for my first operation
- I thought that surgery was an option to consider after I had tried at least some drugs.
- I thought that surgery was an option to consider after I had tried all possible drugs and they had not worked.
- I thought that surgery was an option to consider instead of drugs if I preferred.
  1. **Thinking about your decision to have your first operation to remove the bowel affected by Crohn’s disease,** show how you feel about these statements by circling a number from 1 (strongly agree) to 5 (strongly disagree),

|  | **Strongly agree** | **Agree** | **Neither agree nor disagree** | **Disagree** | **Strongly disagree** |
| --- | --- | --- | --- | --- | --- |
| **It was the right decision** | **1** | **2** | **3** | **4** | **5** |
| **I regret the choice that was made** | **1** | **2** | **3** | **4** | **5** |
| **I would go for the same choice if I had to do it over again** | **1** | **2** | **3** | **4** | **5** |
| **The choice did me a lot of harm** | **1** | **2** | **3** | **4** | **5** |
| **The decision was a wise one** | **1** | **2** | **3** | **4** | **5** |

- 1. **Thinking about the decision to have your first operation to remove the bowel affected by Crohn’s disease, answer these questions** by circling a number from 0 to 9.

| 1. **How much effort was made to help you understand your health issues relating to the Crohn’s disease at the time?** | | | | | | | | | |
| --- | --- | --- | --- | --- | --- | --- | --- | --- | --- |
| **0**  **No effort was made.** | **1** | **2** | **3** | **4** | **5** | **6** | **7** | **8** | **9**  **Every effort was made** |
| 1. **How much effort was made to listen to the things that matter most to you about Crohn’s-related health issues?** | | | | | | | | | |
| **0**  **No effort was made.** | **1** | **2** | **3** | **4** | **5** | **6** | **7** | **8** | **9**  **Every effort was made** |
| 1. **How much effort was made to include what matters most to you in choosing what to do next?** | | | | | | | | | |
| **0**  **No effort was made.** | **1** | **2** | **3** | **4** | **5** | **6** | **7** | **8** | **9**  **Every effort was made** |

- 1. Please answer the following questions about how you feel about your body since your operation(s).

|  | Not at all | A little | Quite a bit | Very much |
| --- | --- | --- | --- | --- |
| Have you been feeling self-conscious about your appearance? | 🞏 | 🞏 | 🞏 | 🞏 |
| Have you felt less physically attractive as a result of your operation? | 🞏 | 🞏 | 🞏 | 🞏 |
| Have you been dissatisfied with your appearance when dressed? | 🞏 | 🞏 | 🞏 | 🞏 |
| Have you been feeling less feminine/masculine as a result of your operation(s)? | 🞏 | 🞏 | 🞏 | 🞏 |
| Do you find it difficult to look at yourself naked? | 🞏 | 🞏 | 🞏 | 🞏 |
| Have you been feeling less sexually attractive as a result of your operation(s)? | 🞏 | 🞏 | 🞏 | 🞏 |
| Do you avoid people because of the way you feel about your appearance? | 🞏 | 🞏 | 🞏 | 🞏 |
| Do you feel the operation(s) has left your body less whole? | 🞏 | 🞏 | 🞏 | 🞏 |
| Have you felt dissatisfied with your body? | 🞏 | 🞏 | 🞏 | 🞏 |

- 1. On a scale from 1 to 7, how satisfied are you with the scar(s) from your operation(s)?

| **Very unsatisfied** |  |  | **Neither unsatisfied nor satisfied** |  |  | **Very satisfied** |
| --- | --- | --- | --- | --- | --- | --- |
| 1 | 2 | 3 | 4 | 5 | 6 | 7 |

- 1. On a scale from 1 to 7, how would you describe your scar(s) from your operation(s)?

| **Very**  **unappealing** |  |  | **Neither unappealing nor pleasing** |  |  | **Pleasing** |
| --- | --- | --- | --- | --- | --- | --- |
| 1 | 2 | 3 | 4 | 5 | 6 | 7 |

- 1. Please score your scar(s) on a scale from 1 (worst possible scar) to 10 (best possible scar).

| 1 | 2 | 3 | 4 | 5 | 6 | 7 | 8 | 9 | 10 |
| --- | --- | --- | --- | --- | --- | --- | --- | --- | --- |

**--------------------------------------------------------------------------------------------------------------**

**This is the end of the questionnaire. Thank you for taking the time to complete it.**

## Table S1. Awareness of surgery as an option

| **Were you aware that surgery was an option for you when treatment options were first discussed with you?*** | **N = 170** |
| --- | --- |
| Yes | 132 (77.6%) |
| No | 38 (22.4%) |
| **How did you first learn about surgery as a treatment option? ^$^** | **N = 169** |
| Gastroenterologist | 114 (67.5%) |
| Surgeon | 62 (36.7%) |
| Nurse specialist | 49 (29.0%) |
| Friends and family | 18 (10.7%) |
| Internet | 18 (10.7%) |
| **Your views on surgery for Crohn’s disease before you were offered an operation^** | **N = 168** |
| “I was not aware that surgery was a treatment option for me before I was offered or listed for my first operation” | 29 (17.3%) |
| “I thought that surgery was an option to consider after I had tried at least some drugs” | 42 (25%) |
| “I thought surgery was an option to consider after I had tried all possible drugs and they had not worked” | 78 (46.4%) |
| “I thought that surgery was an option to consider instead of drugs if I preferred” | 19 (11.3%) |

*Missing data: n=1; ^Missing data: n=3

^$^Totals exceed 171 as patients could report more than one source of information. Missing data: n=2

## Table S2. Surgical outcomes during COVID-19 pandemic versus non-pandemic periods

|  | **Non-pandemic periods** | **Pandemic period*** |  |
| --- | --- | --- | --- |
| **Secondary care vs tertiary units** | 73/117 procedures in a tertiary centre | 37/54 procedures in a tertiary centre | p = 0.437 |
| **Preference for timing of surgery** | 51/117 wished they had surgery sooner, 54/117 felt they had it at the right time | 23/54 wished they had surgery sooner, 26/54 felt they had it at the right time | p = 0.962 |
| **Urgency of operation** | 91/117 planned and 26/117 emergency procedures | 43/54 planned and 11/54 emergency procedures | p = 0.844 |
| **Laparoscopic versus open** | 44/116 laparoscopic and 72/116 open | 25/54 laparoscopic and 29/54 open | p = 0.318 |
| **Stoma formation** | 33/117 procedures with a stoma | 12/54 procedures with a stoma | p = 0.409 |
| **Complication** | 71/116 with no complication | 29/54 with no complication | p = 0.355 |
| **Decision-regret scale score** | Median 5.0 | Median 7.5 | P = 0.644 |

*The pandemic period was considered to last from March 2020 to February 2022 to correspond to UK lockdown restrictions and legal self-isolation requirements
